# Multiple Imputation of Missing Data in Nested Case-Control and Case-Cohort Studies

**DOI:** 10.1111/biom.12910

**Published:** 2018-06-05

**Authors:** Ruth H. Keogh, Shaun R. Seaman, Jonathan W. Bartlett, Angela M. Wood

**Affiliations:** 1 Department of Medical Statistics, London School of Hygiene and Tropical Medicine, London, U.K.; 2 MRC Biostatistics Unit, Cambridge, U.K.; 3 Department of Mathematical Sciences, University of Bath, Bath, U.K.; 4 Department of Public Health and Primary Care, University of Cambridge, Cambridge, U.K.

**Keywords:** Case-cohort study, Cohort study, Cox proportional hazards, Missing data, Multiple imputation, Nested case-control study

## Abstract

The nested case-control and case-cohort designs are two main approaches for carrying out a substudy within a prospective cohort. This article adapts multiple imputation (MI) methods for handling missing covariates in full-cohort studies for nested case-control and case-cohort studies. We consider data missing by design and data missing by chance. MI analyses that make use of full-cohort data and MI analyses based on substudy data only are described, alongside an intermediate approach in which the imputation uses full-cohort data but the analysis uses only the substudy. We describe adaptations to two imputation methods: the approximate method (MI-approx) of White and Royston (2009) and the “substantive model compatible” (MI-SMC) method of Bartlett et al. (2015). We also apply the “MI matched set” approach of Seaman and Keogh (2015) to nested case-control studies, which does not require any full-cohort information. The methods are investigated using simulation studies and all perform well when their assumptions hold. Substantial gains in efficiency can be made by imputing data missing by design using the full-cohort approach or by imputing data missing by chance in analyses using the substudy only. The intermediate approach brings greater gains in efficiency relative to the substudy approach and is more robust to imputation model misspecification than the full-cohort approach. The methods are illustrated using the ARIC Study cohort. Supplementary Materials provide R and Stata code.

## 1 Introduction

Nested case-control and case-cohort studies are the two main designs for a substudy within a cohort. Substudies are used to reduce cost by measuring expensive covariates only on the individuals with the outcome of interest (the “cases”) and a subset of the non-cases. In a nested case-control study each case is matched to a small number of non-cases sampled from the risk set at the case’s event time. In a case-cohort study a random sample of individuals from the full cohort is selected at the start of follow-up—the “subcohort”—and the case-cohort sample is the subcohort plus all the cases from the rest of the cohort. Borgan and Samuelsen (2014) give an overview of these designs. Nested case-control studies are widely used in epidemiology and use of case-cohort studies is increasing (Sharp et al., 2014).

Missing data on covariates are common in observational studies and it is well known that performing an analysis on the subset of individuals with no missing data (a “complete-case analysis”) can result in loss of efficiency and, sometimes, bias in estimated associations. Our focus is on handling missing data in nested case-control and case-cohort studies. Two types of missing data are considered. First, a full cohort with a substudy within it can be considered as having *data missing by design*: the covariates measured only in the substudy are missing by design in the remainder of the cohort. Second, confounders to be adjusted for and which are measured in the full cohort are commonly subject to *data missing by chance*. We make the assumption that data are “missing at random” (MAR) (Seaman et al., 2013).

Multiple imputation (MI) (Rubin, 1987) is a widely used method for handling missing data. We describe how MI methods for full-cohort studies can be adapted to account for the sampling designs of nested case-control and case-cohort studies. We consider three imputation approaches suitable for use in different settings: the “full-cohort,” “intermediate” and “substudy” approaches. For each approach, we consider two MI methods that have been implemented in standard software: the approximate imputation model method of White and Royston (2009), and the “substantive model compatible” method of Bartlett et al. (2015). We also apply the method of Seaman and Keogh (2015) for matched case-control studies to nested case-control studies. Keogh and White (2013) and Borgan and Keogh (2015) used MI to handle data missing by design in nested case-control and case-cohort studies, but did not consider data missing by chance. A major contribution of this article is the extension of MI methods for use in the substudy only.

MI is one way to handle missing data in nested case-control and case-cohort studies. Alternatives are a full maximum likelihood approach (Scheike and Martinussen, 2004; Scheike and Juul, 2004; Borgan and Samuelsen, 2014), an inverse probability weighted (IPW) partial likelihood analysis (Samuelsen, 1997; Kulich and Lin, 2004; Breslow et al., 2009; Støer and Samuelsen, 2013), and targeted maximum likelihood (Rose and van der Laan, 2011). All these alternatives use data from the full cohort from which the substudy is drawn. None has, to our knowledge, considered analysis of just the substudy data or dealt with data missing by chance (as well as by design).

We begin by outlining two MI methods used for Cox regression in full-cohort studies (Section 2). In Section 3, we outline the traditional substudy analyses, discuss what information may be available to the analyst on the full cohort, and propose three approaches. In Sections 4 and 5, we describe adaptations and extensions of MI methods. The methods are assessed using simulation studies (Section 6) and illustrated using data from the Atherosclerosis Risk in Communities (ARIC) Study cohort (The ARIC Investigators, 1989) (Section 7), in which substudies have been frequently used to make use of stored biological samples not processed for the full cohort. In Section 8, we compare our methods with the alternative approaches mentioned above. We conclude with a discussion in Section 9. Supplementary Materials give information about software and example R and Stata code.

## 2 Multiple Imputation for Cox Regression in Full-Cohort Studies

### 2.1 Overview

Let *T* denote the earlier of the event and censoring time, and *D* be an indicator of whether a person had the event (*D* = 1) or was censored (*D* = 0). For simplicity, we describe the methods for a single partially observed covariate *X*, and a vector of fully observed covariates *Z*. See the Supplementary Section S1 for extensions to missingness in several covariates. The hazard function is assumed to be $h\left(t|X,Z\right)=h_{0}\left(t\right)e^{\beta_{X}X+\beta_{Z}^{\prime }Z}.$ The general MI procedure for obtaining estimates of *β_X_* and *β_Z_* is (Carpenter and Kenward, 2013, p. 39):

A model *p*(*X*|*T*, *D*, *Z*; *α_X_*) is specified for *p*(*X*|*T*, *D*, *Z*), the distribution of *X* given *T*, *D* and *Z*. *α_X_* is a vector of regression coefficients and, in the case of a linear regression, the residual variance. Then, for *m* = 1,…, *M*,

(1) The model *p*(*X*|*T*, *D*, *Z*; *α_X_*) is fitted by maximum likelihood to the data on individuals with observed *X*, to obtain an approximate posterior distribution for *α_X_* under a non-informative prior. A value $\alpha_{X}^{\left(m\right)}$ is then drawn from this distribution. For example, if *p*(*X*|*T*, *D*, *Z*; *α_X_*) is a logistic regression model, the posterior for *α_X_* is approximately a normal distribution with mean and variance equal to, respectively, the point and variance estimates from the maximum likelihood fit. See White et al. (2011) for more details.
(2) For each individual *i* with missing *X_i_*, a value $X_{i}^{\left(m\right)}$ is drawn from $p\left(X_{i}|T_{i},D_{i},Z_{i};\alpha_{X}^{\left(m\right)}\right),$ giving a data set (an “imputed” data set) in which there are no missing values.
(3) The substantive model, here the Cox regression model, is fitted to this imputed data set to give estimates $\left({\hat{\beta }}_{X}^{\left(m\right)},{\hat{\beta }}_{Z}^{\left(m\right)}\right)$ of (*β_X_*, *β_Z_*) and ${\hat{\Sigma }}^{\left(m\right)}$ of $\text{Var}\left({\hat{\beta }}_{X}^{\left(m\right)},{\hat{\beta }}_{Z}^{\left(m\right)}\right).$

Estimates $\left({\hat{\beta }}_{X}^{\left(m\right)},{\hat{\beta }}_{Z}^{\left(m\right)}\right)$ (*m* = 1, …, *M*) and ${\hat{\Sigma }}^{\left(m\right)}$ are combined using “Rubin’s rules” (Rubin, 1987) to give an overall estimate of (*β_X_*, *β_Z_*) and corresponding variance-covariance matrix. Although in principle any model *p*(*X*|*T*, *D*, *Z*; *α_X_*) could be used, potentially serious bias in the estimates of (*β_X_*, *β_Z_*) and their variance-covariance matrix could arise if the imputation model is mis-specified. In particular, if the imputation model is not compatible with the Cox (substantive) model that is used for the analysis (which is assumed to be correctly specified), this may imply the former is mis-specified (Bartlett et al., 2015). We now describe two methods that have been proposed for imputing from a model *p*(*X*|*T*, *D*, *Z*; *α_X_*) that is either exactly or approximately compatible with a Cox model.

### 2.2 Approximate Imputation Method (“MI-approx”)

White and Royston (2009) derived an approximate form for *p*(*X*|*T*, *D*, *Z*; *α_X_*) that resulted in an imputation model for *X* that includes *Z*, *D* and $\hat{H}\left(T\right)$ (the Nelson–Aalen estimate of the marginal cumulative hazard) as predictors. For continuous *X*, the imputation model is $X=\alpha_{0}+\alpha_{1}^{\prime }Z+\alpha_{2}D+\alpha_{3}\hat{H}\left(T\right)+\epsilon ,$ where *ϵ* is a normally distributed residual. For binary *X*, the imputation model is logit $\left\{Pr\left(X=1|Z,D,T\right)\right\}=\alpha_{0}+\alpha_{1}^{\prime }Z+\alpha_{2}D+\alpha_{3}\hat{H}\left(T\right).$

### 2.3 Substantive Model Compatible Imputation Method (“MI-SMC”)

MI-approx has been found to work well in a range of circumstances (White and Royston, 2009; Carpenter and Kenward, 2013). However, it can perform badly when there are large effect sizes and/or when the event rate is high (White and Royston, 2009). Also, the approximation is derived assuming that *X* has only a main linear effect in the Cox model. Thus in cases where the substantive model contains higher-order effects or interactions, using MI-approx can lead to bias (Seaman et al., 2012). Bartlett et al. (2015) described the “substantive model compatible” (SMC) MI method for producing imputations from a model that is compatible with the user’s chosen substantive model. They use a rejection sampling algorithm, in which a potential value for *X* is sampled from a “proposal distribution” *p*(*X*|*Z*; *γ_X_*) and then a “rejection rule” is used to determine whether this proposed value is accepted as a draw from *p*(*X*|*T*, *D*, *Z*). Details are given in Supplementary Section S2.

## 3 Missing Data in Nested Case-Control and Case-Cohort Studies

To illustrate the issues, we introduce new notation to be used throughout the rest of the article. The covariates of interest are (*X*_1_, *X*_2_, *Z*), where *X*_1_ are subject to data missing by design, *X*_2_ are subject to data missing by chance, and *Z* are observed in the full cohort with no missing values. *X*_1_ was intended to be measured only in the substudy, and is missing in the remainder of the full cohort. *X*_2_ was intended to be measured on all individuals in the full cohort but has some values missing by chance. *X*_1_ may also have some values missing by chance within the substudy; for simplicity of the explanations, we do not consider that situation here, though all of the methods accommodate missingness by chance in *X*_1_. The hazard function for the population of interest is assumed to be $h\left(t|X_{1},X_{2},Z\right)=h_{0}\left(t\right)\text{exp}\left(\beta_{X1}^{\prime }X_{1}+\beta_{X2}^{\prime }X_{2}+\beta_{Z}^{\prime }Z\right).$

Let *τ*_1_, *τ*_2_,…, *τ_J_* denote the unique event times, assuming no ties for simplicity. The analysis of the full-cohort data would use the partial likelihood, $L_{\text{full}-\text{cohort}}=\prod_{j=1}^{J}\frac{e^{\beta_{X1}^{\prime }X_{1i_{j}}+\beta_{X2}^{\prime }X_{2i_{j}}+\beta_{Z}^{\prime }Z_{i_{j}}}}{{\Sigma_{k\in R_{j}}}^{e^{\beta_{X1}^{\prime }X_{1k}+\beta_{X2}^{\prime }X_{2k}+\beta_{Z}^{\prime }Z_{k}}}},$ where *i*_j_ is the index of the individual who has the event at time *τ_j_* (*j* = 1,…, *J*) and *R_j_* denotes the set of individuals at risk at time *τ_j_*. Nested case-control and case-cohort studies are analysed using the modified partial likelihood $L_{\text{substudy}}=\prod_{j=1}^{J}\frac{e^{\beta_{X1}^{\prime }X_{1i_{j}}+\beta_{X2}^{\prime }X_{2i_{j}}+\beta_{Z}^{\prime }Z_{i_{j}}}}{{\Sigma_{k\in {\tilde{R}}_{j}}}^{e^{\beta_{X1}^{\prime }X_{1k}+\beta_{X2}^{\prime }X_{2k}+\beta_{Z}^{\prime }Z_{k}}}},$ where ${\tilde{\text{R}}}_{j}$ denotes a subset of the full risk set *R_j_*. In a nested case-control study ${\tilde{\text{R}}}_{j}$ consists of the case at time *τ_j_* plus the set of *c*(*τ_j_*) controls sampled from the risk set at that time (*c*(*τ_j_*) is usually constant). In a case-cohort study, ${\tilde{\text{R}}}_{j}$ denotes the set of individuals in the sub-cohort who are at risk at time *τ_j_*, plus the case occurring at time *τ_j_* if that case is outside the subcohort. In a nested case-control study estimation is by maximum partial likelihood. In a case-cohort study, *L*_substudy_ is a pseudo–partial likelihood and a sandwich estimator or appropriate alternative should be used for standard errors (Borgan and Samuelsen, 2014).

Analysts working with nested case-control and case-cohort studies may have access to different amounts of information about the full cohort. In a “maximum information setting” all the observed data on the full cohort are available, meaning that the analyst knows (*T*, *D*) for each individual in the full cohort and the values of *X*_1_, *X*_2_, and *Z* for all individuals in the full cohort on whom they are observed. In a “minimum information setting” the analyst has only the substudy data. A third possibility, referred to as the “time-only information setting”, is that the analyst has the information on (*T*, *D*) in the full cohort and only the observed data on (*X*_1_, *X*_2_, *Z*) in the substudy. In Sections 4 and 5, we describe MI methods for three different approaches—the “full-cohort approach”, the “intermediate approach”, and the “substudy approach”—and discuss their suitability for these settings.

## 4 Full-Cohort and Intermediate Approaches

The full-cohort approach is suitable for the maximum information setting: missing values in *X*_1_ and *X*_2_ are imputed using either the MI-approx or MI-SMC method from Section 2 and the Cox model is fitted to the full cohort. Keogh and White (2013) used MI-approx and a version of MI-SMC, showing that an MI analysis using full cohort data can lead to important gains in efficiency compared with the traditional substudy analysis. Further investigations using MI-approx were performed for nested case-control studies by Borgan and Keogh (2015). However, this earlier work focused on data missing by design.

There may be a reluctance to fit the substantive model to data in which a large proportion of *X*_1_ values have been imputed, for fear that the imputation model might be misspecified. An alternative is to perform both imputation and analysis on just the substudy data. This is the “substudy approach” described in Section 5. An intermediate approach is to perform imputation on the full cohort but then fit the substantive model to just the substudy data. This would be expected to be more efficient than the substudy approach and more robust to misspecification of the imputation model than the full-cohort approach. The full-cohort and intermediate approaches assume that data on (*T*, *D*, *X*_1_, *X*_2_, *Z*) in the full cohort are MAR. This is true if missingness by chance in *X*_2_ depends only on observed variables (*T*, *D*, *Z*), as missingness by design in *X*_1_ depends only on (*T*, *D*), which are observed in this setting.

## 5 Substudy Approach

The methods we now describe impute only the missing data in the substudy and are suitable for use in the time-only information setting and, in some cases, in the minimum information setting. They can also be applied in the maximum information setting by discarding the data on either (*X*_2_, *Z*) or (*T*, *D*, *X*_2_, *Z*) for individuals not in the substudy. *X*_1_ and *Z* are fully observed in the substudy and there are data missing by chance in *X*_2_. The task is therefore to impute *X*_2_. Missing *X*_2_ values should be drawn from *p*(*X*_2_|*T*, *D*, *X*_1_, *Z*, *S* = 1), where S=1 denotes that an individual belongs to the substudy. Since the probability of being sampled for the substudy depends only on (*T*, *D*), *p*(*X*_2_|*T*, *D*, *X*_1_, *Z*, *S* = 1) = *p*(*X*_2_|*T*, *D*, *X*_1_, *Z*). This justifies the following adapted versions of MI-approx and MI-SMC when the data on (*T*, *D*, *X*_1_, *X*_2_, *Z*) in the substudy are MAR, that is, when missingness by chance in *X*_2_ in the substudy depends only on observed variables ((*T*, *D*, *X*_1_, *Z*) in this setting).

### 5.1 MI-Approx

The MI-approx method of Section 2.2 can be immediately applied just to the substudy once an estimate of *H*(*t*), the cumulative hazard in the population, has been obtained. If data on (*T*, *D*) are available for the full cohort (maximum and time-only information settings), then the Nelson–Aalen estimate of *H*(*t*) can be obtained easily. If data on (*T*, *D*) are not available for the full cohort (minimum information setting), *H*(*t*) must be estimated using only the substudy data. In a nested case-control study there is no representative sample from the full cohort, and so it is not possible to estimate *H*(*t*) in the minimum information setting. In a case-cohort study an unbiased estimate could be obtained by applying the Nelson–Aalen estimator to the subcohort only. However, this is inefficient because it does not use cases outside the subcohort. A weighted estimator that uses information on events occurring outside the subcohort and the subcohort sampling fraction is ${\hat{H}}_{\text{CC}}(t)=\frac{n_{S}(0)}{n}\sum_{\tau_{k}\leq t}\frac{d(\tau_{k})}{n_{S}(\tau_{k})}$ where *d*(*τ_k_*) is the number of events at time *τ_k_* (in our setting, *d*(*τ_k_*) = 1), *n_S_* (*τ_k_*) is the number at risk in the subcohort at time *τ_k_*, and *n* is the total number in the cohort. This uses the assumption that the ratio of the number at risk in the full cohort to the number at risk in the subcohort is approximately constant over time (*n/n_S_*(0)). Notice that ${\hat{H}}_{\text{CC}}(t)$ is proportional to $\sum_{\tau_{k}\leq t}\frac{d(\tau_{k})}{n_{S}(\tau_{k})},$ which is approximately equal to ${\hat{H}}_{\text{CC}}^{*}(t)=\sum_{\tau_{k\leq t}}\frac{d(\tau_{k})}{n_{cc}(\tau_{k})},$ where *n_cc_*(*τ_k_*) = *n_S_*(*τ_k_*) if the case whose event time is *τ_k_* is in the subcohort and *n_cc_*(*τ_k_*) = *n_S_*(*τ_k_*) + 1 otherwise. This is a useful result, because ${\hat{H}}_{CC}^{*}(t)$ can be obtained directly by applying the Nelson–Aalen estimator to the case-cohort data.

### 5.2 MI-SMC

As explained in Supplementary Section S2, MI-SMC involves estimating *p*(*X*_2_ | *X*_1_, *Z*) and the (population) baseline cumulative hazard, *H*_0_(*t*). The distribution of *X*_2_ given (*X*_1_, *Z*) and *S* = 1 and the baseline cumulative hazard given *S* = 1 will typically differ from *p*(*X*_2_ | *X*_1_, *Z*) and *H*_0_(*t*). For this reason, modified estimators of these latter quantities are needed when applying MI-SMC to just the substudy. In a case-cohort study *H*_0_(*t*) can be estimated using the modified Breslow estimator (Borgan and Samuelsen, 2014) ${\hat{H}}_{0}^{\text{CC}}(t)=\frac{n_{S}(0)}{n}\sum_{\tau_{k}\leq t}\frac{1}{\sum_{l\in S_{k}}\text{exp}({\hat{\beta }}_{X1}X_{1l}+{\hat{\beta }}_{X2}X_{2l}+{\hat{\beta }}_{Z}Z_{l})},$ where *S_k_* denotes the set of subcohort members who are at risk at time *τ_k_*. This uses the subcohort sampling fraction *n_S_*(0)/*n*, which is typically known. The model for *p*(*X*_2_ | *X*_1_, *Z*) can be fitted to the subcohort (because it is a random sample from the full cohort).

In a nested case-control study, *H*_0_(*t*) can be estimated using (Langholz and Borgan, 1997) ${\hat{H}}_{0}^{\text{NCC}}(t)=\sum_{\tau_{k}\leq t}\frac{1}{\sum_{l\in {\tilde{R}}_{k}}\left\{n(\tau_{k})/(c(\tau_{k})+1)\}\text{exp}({\hat{\beta }}_{X1}X_{1l}+{\hat{\beta }}_{X2}X_{2l}+{\hat{\beta }}_{Z}Z_{l}\right)},$ where *n*(*τ_k_*) is the number at risk in the full cohort at time *τ_k_* and *c*(*τ_k_*) is the number of controls selected for the case at time *τ_k_*. This requires information on (*T, D*) in the full cohort and can therefore be used in the maximum and time-only information settings. There are two ways of estimating the parameters of *p*(*X*_2_|*X*_1_, *Z*). The first is to assume that the outcome is rare, so that *p*(*X*_2_|*X*_1_, *Z*) ≈ *p*(*X*_2_|*X*_1_, *Z*, *D* = 0). *p*(*X*_2_|*X*_1_, *Z*) can then be estimated using just the controls. The second uses all the individuals in the nested case-control sample to fit *p*(*X*_2_|*X*_1_, *Z*) via inverse probability weighted (IPW) estimation. Non-cases are weighted by the reciprocal of their probability of ever being sampled as a control. For individual *i* with right-censoring time *T_i_*, this probability is (Samuelsen, 1997) $1-\prod_{j=1}^{J}I(\tau_{j}\leq T_{i})\left(1-\frac{c(\tau_{j})}{n(\tau_{j})-1}\right).$

### 5.3 “MI Matched Set”: An Alternative for Nested Case-Control Studies

We now outline a method that can be used for nested case-control studies in the minimum information setting. Nested case-control studies are a type of matched case-control study and the traditional partial likelihood analysis is equivalent to conditional logistic regression. Seaman and Keogh (2015) described two MI methods for missing data in matched studies analysed using conditional logistic regression. We apply their “MI matched set” method to nested case-control studies. This involves imputing the missing data on each individual in a given set using the data on that individual and all the other individuals in the same set, as follows. Assuming that the same number of controls is selected for each case, let {*X*_1*jk*_, *X*_2*jk*_, *Z_jk_*} (*k* = 1,…, *K*) denote the explanatory variables for the *k*th individual in the sampled risk set at event time *τ_j_*, where the data are arranged so that {*X*_1*j*1_, *X*_2*j*1_, *Z*_*j*1_} denote the values for the individual who is the case at time *τ_j_* and {*X*_1*jk*_, *X*_2*jk*_, *Z_jk_*} (*k* = 2,…, *K*) are the values for the individuals sampled as controls at that time. The imputation model for *X*_2*jk*_ (*k* = 1,…, *K*) includes *X*_1*jk*_, *Z_jk_*, ∑_*l*≠*k*_ *X*_1*jl*_, ∑_*l*≠*k*_ *X*_2*jl*_, and ∑_*l*≠*k*_ *Z_jl_*. Variable numbers of controls per case can be accommodated (Seaman and Keogh, 2015).

## 6 Simulation Study

### 6.1 Simulating the Data

Data were generated for full cohorts of 15,000 individuals. We consider three correlated explanatory variables: *X*_1_ (normally distributed), *X*_2_ (binary), and *Z* (binary). Event times were generated from a Weibull hazard: *h*(*t*|*X*_1_, *X*_2_, *Z*) = *tλ* exp {*β*_*X*1_*X*_1_ + *β*_*X*2_*X*_2_ + *β_Z_Z*}. We considered values *β_X_*_1_ = *β_X_*_2_ = *β_Z_* = 0.2 or 0.7. From each cohort, we sampled a case-cohort study using a subcohort of 750 individuals, and a nested case-control study with 1 or 4 controls for each case. Z is fully observed in the full cohort. *X*_1_ is observed only in the substudy. Missingness by chance was generated in *X*_2_ for 10% or 50% of individuals in the full cohort, with the probability of missingness depending on *Z* and *D* and their interaction. Full details are in Supplementary Section S3. 1000 simulated data sets were generated.

### 6.2 Analyses Performed

In each simulated data set, we performed a full-cohort analysis and standard substudy analysis before introducing the missing data. These are the “complete-data” analyses. After introducing missing data a complete-case analysis was performed on the substudy, omitting individuals with data missing by chance in *X*_2_. Analyses were performed using the full-cohort, intermediate and substudy approaches. Table 1 summarizes the analyses. For the MI-approx substudy approach used for a case-cohort study, we present results using ${\hat{H}}_{\text{CC}}^{*},$ since differences between results obtained using the three different estimates described in Section 5.1 were negligible and ${\hat{H}}_{\text{CC}}^{*}$ is arguably the simplest to obtain. MI methods used 10 imputed data sets.

**Figure 1:**
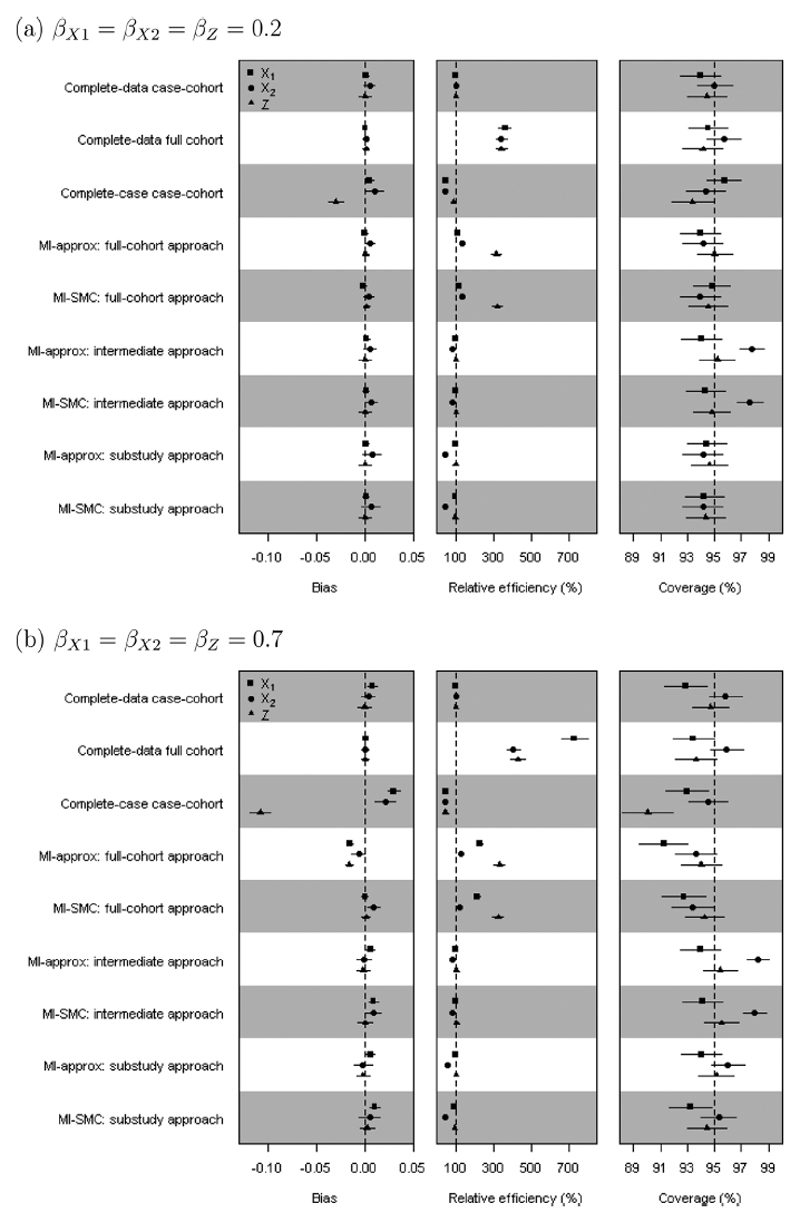
Simulation study results: case-cohort study within a cohort with 50% missing *X*_2_. The points are the means of the point estimates from 1000 simulated data sets. Horizontal lines around each point are the 95% confidence intervals obtained based on Monte Carlo errors. The relative efficiency is relative to the complete-data substudy analysis.

**Figure 2:**
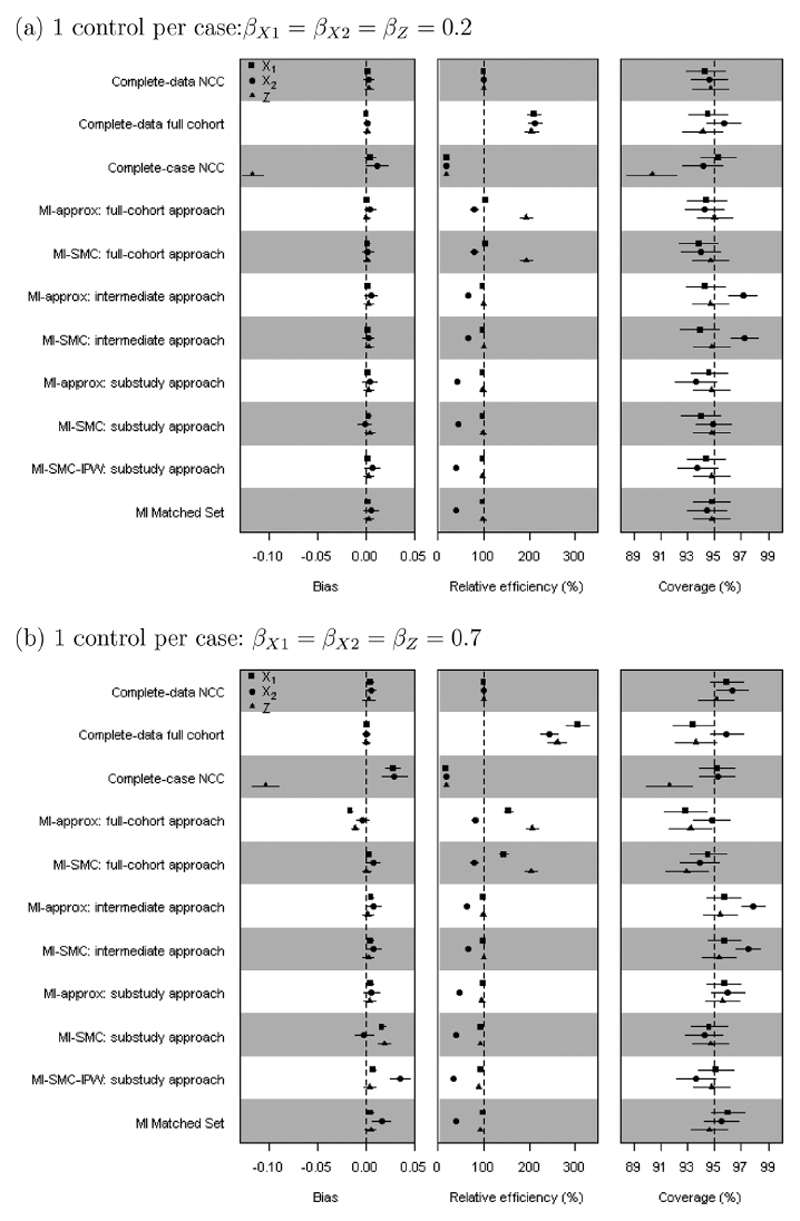
Simulation study results: nested case-control (NCC) study with one control per case within a cohort with 50% missing *X*_2_. The points are the means of the point estimates from 1000 simulated data sets. Horizontal lines around each point are the 95% confidence intervals obtained based on Monte Carlo errors. The relative efficiency is relative to the complete-data substudy analysis.

We summarize the results for *β*_*X*1_, *β*_*X*2_, and *β_Z_* in terms of bias, coverage of 95% confidence intervals, and efficiency relative to the complete-data substudy analysis (ratio of empirical variances from the complete-data substudy analysis and the comparison analysis). Figures 1 and 2 show results from the simulation scenarios with 50% missing data in *X*_2_, and for 1 control per case in the nested case-control study. Results from other scenarios are shown in Supplementary Figures S1–S4.

### 6.3 Results: Case-Cohort Study

As expected, the complete-data full-cohort analysis and complete-data case-cohort analysis give approximately unbiased estimates, though for large effect sizes (Figure 1b) there is some small finite-sample bias in the estimate for *β*_*X*1_. The complete-data full-cohort analysis gives correct coverage. The same is true for the complete-data case-cohort analysis, except for *β_X_*_1_ when the effect size is large; then there is minor under-coverage. The complete-case case-cohort analysis gives biased estimates, particularly for *β_Z_*, but also for *β_X_*_1_ and *β_X_*_2_ when the effect size is large. There is also below nominal level coverage.

In the full-cohort approach, both MI-approx and MI-SMC give unbiased estimates for small effect sizes. MI-SMC also gives unbiased estimates for larger effect sizes, while MI-approx gives minor bias towards the null due to the approximations used, leading to some under-coverage, especially for *β*_*X*_1__. Previous investigations of MI-approx have made similar findings, which are due to the approximate nature of this MI method (White and Royston, 2009). Standard errors (SEs) from both methods are approximately unbiased, but for larger effects sizes MI-SMC tends slightly to underestimate SEs, leading to slight under-coverage. The full-cohort approach gives large efficiency gains for all three parameters relative to the complete-data substudy analysis. The gain is greatest for *β_Z_*, because Z is observed in the full cohort. There is also a substantial gain in efficiency for *β_X_*_1_ when the effect size is large.

For small effect size the substudy approach, using both MI-approx and MI-SMC, gives unbiased estimates, correct SEs and good coverage. For larger effect sizes, we again see approximately unbiased estimates, though there is small bias away from the null for *β*_*X*_1__ (as in the complete-data substudy analysis), which we attribute to finite sample bias. The SE for *β_X_*_1_ is slightly underestimated and there is slight under-coverage, again as in the complete data case-cohort analysis.

The intermediate approach, using both MI-approx and MI-SMC, gives approximately unbiased estimates for small and large effect sizes and large gains in efficiency for all three parameters relative to the complete-case analysis, bringing the efficiency close to 100% relative to the complete-data substudy analysis, although the efficiency gains are not as substantial as under the full-cohort approach. The SE for *β*_*X*2_ tends to be over estimated, resulting in slight over-coverage for this parameter. This is a situation in which the imputer is assuming that certain associations between variables are the same in the full cohort as they are in the substudy, and therefore assuming more than the analyst, which is known to result in Rubin’s Rule for the variance overestimating the SEs (Robins and Wang, 2000).

Across all the MI analyses, results from MI-approx are very similar to the corresponding results from MI-SMC. An exception is the MI analysis using the full cohort when the effect sizes are large; here the MI-approx estimates are slightly biased towards the null.

We observed under-coverage from several methods, including the complete-data analysis, when the effect size is large. This is believed to be due to finite sample size. In sensitivity analyses, we increased the size of the cohort or the relative size of the subcohort (Supplementary Figure S5). With a larger cohort the coverage for the complete-data full-cohort and complete-data case-cohort analysis was improved. For the full-cohort approach using MI-SMC the coverage improved as the relative size of the subcohort increased. Increasing the sample size or the relative subcohort size did not reduce the bias in estimates from the full-cohort approach using MI-approx when the effect size is large. Increasing the sample size or the relative subcohort size improved the coverage from the substudy approach using MI-SMC and MI-approx.

### 6.4 Results: Nested Case-Control Study

The relative performances of methods used for nested case-control studies are similar to what was found for case-cohort studies (Figure 2, Supplementary Figure S2). MI matched set also gives approximately unbiased estimates and good coverage, with the SEs being similar to those using MI-approx and MI-SMC in the substudy approach. When there are 4 controls per case (Supplementary Figures S3 and S4) there is less to be gained by using the full-cohort data, though the gains are still substantial. In MI-SMC using the substudy approach, fitting the covariate model *p*(*X*_2_|*X*_1_, *Z*) using the controls performed slightly better than fitting it using the IPW method described in Section 5.2.

### 6.5 Additional Simulation Scenario: Model Misspecification

We investigated the performance of the methods under imputation model misspecification, focussing on case-cohort studies. Other aspects of the simulations were as above with *β_X_*_1_ = *β*_*X*2_ = *β_Z_* = 0.7. *X*_1_ was generated from a log normal distribution with log *X*_1_ having mean 0.25*Z* and standard deviation 0.65. *X*_2_ was generated using logit $\text{Pr}(X_{2}=1|X_{1},Z)=0.5Z+0.25(X_{1}+X_{1}^{2}).$ In MI-approx, we used a misspecified normal imputation model for *X*_1_ and a misspecified logistic imputation model for *X*_2_. MI-SMC allows imputed variables to be used on a transformed scale in the substantive model. We considered two forms for the model for *X*_1_|*X*_2_, *Z*, one correctly specified and one misspecified. In both cases, we used a misspecified model for *X*_2_|*X*_1_, *Z*. See Supplementary Section S4 for details.

**Figure 3:**
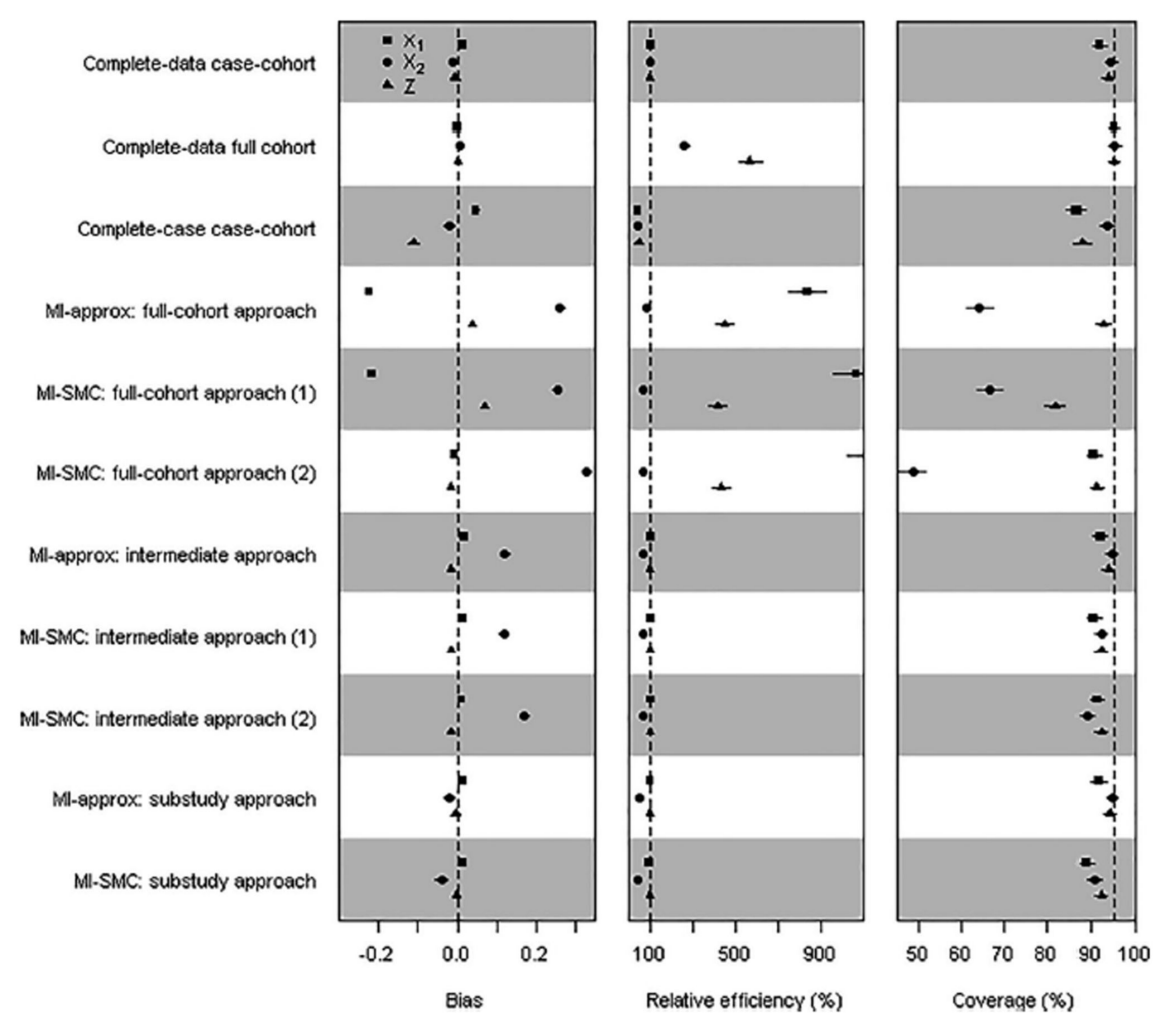
Simulation study results from the additional scenario in which the imputation model is misspecified: case-cohort study within a cohort with *β_X_*_1_ = *β_X_*_2_ = *β_Z_* = 0.7 and 50% missing *X*_2_. In “MI-SMC full-cohort approach (1)” and “MI-SMC intermediate approach (1)” the model used for the proposal distribution *p*(*X*_1_|*X*_2_*, Z*) was a normal distribution (with main effects of *X*_2_ and *Z*) and in “MI-SMC full-cohort approach (2)” and “MI-SMC intermediate approach (2)” the model used for the proposal distribution for *p*(log *X*_1_|*X*_2_*, Z*) was a normal distribution (with main effects of *X*_2_ and *Z*). In both (1) and (2), and in “MI-SMC substudy approach,” the model used for the proposal distribution *p*(*X*_2_|*X*_1_*, Z*) was a logistic regression with main effects of *X*_1_ and *Z*. The horizontal lines around each point are the 95% confidence intervals obtained based on Monte Carlo errors. Coverage for *X*_1_ in “MI-approx full-cohort approach” and “MI-SMC full-cohort approach (1)” is 0% and not shown on the plot.

Results are shown in Figure 3. The impact of using misspecified imputation models is severe under the MI full-cohort approach: there is large bias and poor coverage, especially for *β*_*X*_1__. The MI-SMC analyses that use a correctly specified model in the proposal distribution for *X*_1_ perform better, demonstrating the important feature of this imputation method that it allows a transformed covariate to be imputed assuming that it follows a conditional normal distribution, but with the untransformed covariate entering the substantive model linearly. As expected, the intermediate approach is less affected by the imputation model misspecification, particularly for *β*_*X*_1__. The substudy approach performs well despite the misspecification of the imputation model for *X*_2_, with only small bias in *β*_*X*_2__.

## 7 Application to the ARIC Study Cohort

We applied the methods to data from the ARIC Study cohort to investigate the association between death due to cardiovascular disease and the traditional risk factors of systolic blood pressure (mmHG), total and HDL cholesterol (mmol/l), smoking and body mass index (BMI), with adjustment for sex, age, race, and education level. The cohort comprises 15,792 individuals and there were 1089 cardiovascular deaths over the course of follow-up. We created a case-cohort study by sampling a subcohort of 650 individuals, and a nested case-control study with one control per case, reflecting real examples (Atherosclerosis Risk in Communities Study, 2011; Sanders et al., 2015). Measurements of total and HDL cholesterol were set to be missing outside of the substudy, creating missingness by design. Missingness by chance was introduced in systolic blood pressure, smoking status, body mass index, race, and education level; the probability of missingness depended on sex and age, and we generated 10% missing data conditionally independently in each variable in the full cohort.

Age, systolic blood pressure, total cholesterol, HDL cholesterol and BMI are continuous variables and were assumed to have linear effects on the log hazard. The remaining variables were treated as categorical. Results are shown in Table 2. In the case-cohort setting the log hazard ratio estimates are broadly similar across the different analyses. The MI full-cohort approach gives SEs that are typically less than half those obtained in the complete-case analysis, with the gain in efficiency being somewhat less for the two cholesterol variables, which are missing by design in the full cohort. The MI substudy and intermediate approaches give smaller but still substantial gains in efficiency. The results are similar in the nested case-control setting. Using MI matched set gives results similar to those obtained using MI-approx and MI-SMC under the substudy approach. The following explanatory variables are statistically significantly associated with an increased hazard for CVD death (*p<* 0.05) in all analyses: being male, older age, smoking, non-white race, higher BMI. The following additional explanatory variables are statistically significantly associated with an increased hazard in the complete-data full cohort analysis and in all MI analyses, but not in the complete-case analyses: lower level of education (not in MI-approx and MI-SMC in the substudy and intermediate approaches), higher SBP, lower HDL cholesterol.

## 8 Alternative Approaches

In the maximum information setting, our full-cohort approach using MI followed by a partial likelihood analysis is one way of making efficient use of data available on the full cohort. An alternative is a full maximum likelihood approach, which incorporates full cohort data by considering the full cohort and the substudy as a two-phase design in which some covariates are observed in the phase-one sample (the full cohort) and other expensive covariates are observed only in the phase-two sample (the substudy). An overview is given by Borgan and Samuelsen (2014). The full likelihood involves the conditional distribution of the phase-two covariates conditional on the phase-one covariates (*X*_1_|*X*_2_*, Z*). Scheike and Martinussen (2004) and Scheike and Juul (2004) described this method in the context of case-cohort and nested case-control studies respectively, using a non-parametric approach in which the phase-one covariates are discrete and the conditional distribution has point masses at the observed covariate values. Estimation is via the EM Algorithm. Zeng and Lin (2014) presented a similar method which accommodates continuous phase-one covariates via an approximation. The full maximum likelihood methods require assumptions about the distribution of phase-two covariates conditional on phaseone covariates. This is similar to the modeling assumptions required for the conditional covariate distributions used in MI-SMC. In some situations MI-SMC should be as efficient as a full maximum likelihood analysis. There does not appear to exist any ready-made software for implementing the full maximum likelihood methods.

Another general approach described for making efficient use of data in two-phase studies uses an inverse probability weighted (IPW) partial likelihood. Full-cohort information is used only in the construction of the weights. Kulich and Lin (2004) and Breslow et al. (2009) described this for case-cohort studies. IPW approaches for nested case-control studies were described by Samuelsen (1997) and Støer and Samuelsen (2013). These IPW methods have been found to be less efficient then the MI approach (Keogh and White, 2013; Borgan and Keogh, 2015). The IPW methods are closely related to developments for handling of missing data in full cohort studies analysed using Cox regression (Robins et al., 1994). Rose and van der Laan (2011) proposed an approach to using full-cohort data in nested case-control and case-cohort studies by using targeted maximum likelihood estimation (TMLE); this does not appear to have been used in practice and is not based on widely familiar concepts.

As noted earlier, none of the methods mentioned above have, to our knowledge, considered data missing by chance as well as data missing by design, though extensions to that would likely be possible. Nor have there been previous methods designed for handling of missing data in the sub-study only. Several methods have been described for handling missing data in matched case-control studies, as summarized in the introduction of Seaman and Keogh (2015), some of which could potentially be extended for nested case-control studies.

## 9 Discussion

We have shown how to use MI to handle missing covariate data in case-cohort and nested case-cohort studies, including data missing by design and missing by chance. We adapted the methods of White and Royston (2009) (MI-approx) and Bartlett et al. (2015) (MI-SMC), and applied a method for matched case-control studies (Seaman and Keogh, 2015) (MI matched set) to nested case-control studies. All the methods described can be applied using readily available software, including new additions we have made to the smcfcs package in R. See Supplementary Section S5. Several extensions to accommodate covariate-dependent censoring, left-truncation, nested case-control studies with additional matching and case-cohort studies with stratified subcohort sampling depending on *Z* are described in Supplementary Section S6.

Our simulations show that MI works well at handling missing data in case-cohort and nested case-control studies when the imputation models are (approximately) correctly specified. Relative to a complete-case substudy analysis, substantial gains in efficiency as well as bias-correction are possible by making use of full-cohort data. An intermediate approach, in which the imputation is performed on the full cohort but the substantive model is fitted only in the substudy, is more robust to imputation model misspecification than the full cohort approach. For data missing by chance in the substudy, gains in efficiency and bias-correction are possible by applying MI to the substudy only. MI matched set works well for nested case-control studies, and does not require full-cohort information. MI-approx and MI-SMC both performed well in the settings considered. An advantage of MI-SMC over MI-approx is that it allows variable transformations and interactions in the substantive model.

Several non-MI methods have been described for making use of full cohort data in case-cohort and nested case-control studies in Section 8, and a detailed comparison is merited, particularly to investigate the impact of model misspecfication. A major advantage of MI is that it can be applied in standard software and is familiar to many researchers.

## Supplementary Material

A Web Appendix, referenced in Sections 1, 2.1, 2.3, 5.2, 6.1–6.5, and 9 is available with this article at the *Biometrics* website on Wiley Online Library. Example R and Stata code for applying the methods described in this article is available at https://github.com/ruthkeogh/MI-CC. We have implemented the MI-SMC substudy approach in the smcfcs package in R (https://github.com/jwb133/smcfcs, and also available on CRAN).

Supplementary information

## Figures and Tables

**Table 1:** Analyses performed in each simulated data set. NCC indicates “nested case-control.”

Analysis	Variables with missing data	Imputation	Analysis
	**Case-cohort sample within a full cohort**		
Complete-data case-cohort	NA	NA	Case-cohort
Complete-data full cohort	NA	NA	Full cohort
Complete-case case-cohort	*X*_2_	NA	Case-cohort
MI-approx: full-cohort approach	*X*_1_, *X*_2_	Full cohort	Full cohort
MI-SMC: full-cohort approach	*X*_1_, *X*_2_	Full cohort	Full cohort
MI-approx: intermediate approach	*X*_1_, *X*_2_	Full cohort	Case-cohort
MI-SMC: intermediate approach	*X*_1_, *X*_2_	Full cohort	Case-cohort
MI-approx: substudy approach	*X*_2_	Case-cohort	Case-cohort
MI-SMC: substudy approach	*X*_2_	Case-cohort	Case-cohort
	**Nested case-control sample within a full cohort**		
Complete-data NCC	NA	NA	NCC
Complete-data full cohort	NA	NA	Full cohort
Complete-case NCC	*X*_2_	NA	NCC
MI-approx: full-cohort approach	*X*_1_, *X*_2_	Full cohort	Full cohort
MI-SMC: full-cohort approach	*X*_1_, *X*_2_	Full cohort	Full cohort
MI-approx: intermediate approach	*X*_1_, *X*_2_	Full cohort	NCC
MI-SMC: intermediate approach	*X*_1_, *X*_2_	Full cohort	NCC
MI-approx: substudy approach	*X*_2_	NCC	NCC
MI-SMC: substudy approach	*X*_2_	NCC	NCC
MI matched set	*X*_2_	NCC	NCC

**Table 2:** Results from applying MI methods to the ARIC cohort with case-cohort and nested case-control substudies, to investigate the association between the variables listed and the hazard for death due to cardiovascular disease. The estimates are log hazard ratios. [*Education levels: 1 - primary, 2 - Secondary, 3 - Vocational/University].

(a) Case-cohort study within the full cohort								

	Complete-data full cohort	Complete-case	MI full-cohort approach		MI substudy approach		MI intermediate approach	
					
			MI-approx	MI-SMC	MI-approx	MI-SMC	MI-approx	MI-SMC
	Est (SE)	Est (SE)	Est (SE)	Est (SE)	Est (SE)	Est (SE)	Est (SE)	Est (SE)

Sex (baseline=male)	−0.481 (0.073)	−0.796 (0.188)	−0.436 (0.072)	−0.453 (0.071)	−0.508 (0.139)	−0.481 (0.140)	−0.482 (0.140)	−0.502 (0.140)
Age (years)	0.097 (0.006)	0.091 (0.017)	0.095 (0.006)	0.095 (0.006)	0.082 (0.012)	0.082 (0.012)	0.083 (0.012)	0.083 (0.012)
Education*: 1	ref						
Education: 2	−0.149 (0.092)	0.323 (0.274)	−0.119 (0.096)	−0.090 (0.101)	0.066 (0.208)	0.026 (0.214)	0.062 (0.214)	0.052 (0.218)
Education: 3	−0.426 (0.097)	0.136 (0.288)	−0.373 (0.101)	−0.369 (0.103)	−0.093 (0.211)	−0.097 (0.215)	−0.080 (0.221)	−0.114 (0.233)
Non-smoker	ref							
Current smoker	0.641 (0.067)	0.735 (0.178)	0.609 (0.071)	0.622 (0.070)	0.584 (0.140)	0.609 (0.141)	0.595 (0.143)	0.610 (0.141)
White race	ref							
Non-white race	0.434 (0.072)	0.456 (0.201)	0.508 (0.077)	0.498 (0.080)	0.363 (0.159)	0.319 (0.159)	0.365 (0.161)	0.355 (0.166)
SBP (mmHG)	0.015 (0.002)	0.008 (0.004)	0.015 (0.002)	0.015 (0.002)	0.012 (0.004)	0.013 (0.003)	0.013 (0.004)	0.013 (0.004)
BMI (kg/metres^2^)	0.040 (0.006)	0.060 (0.019)	0.037 (0.006)	0.038 (0.006)	0.042 (0.014)	0.042 (0.014)	0.040 (0.014)	0.042 (0.014)
Total chol. (mmol/l)	0.055 (0.030)	0.055 (0.066)	0.037 (0.043)	0.043 (0.050)	0.026 (0.052)	0.028 (0.052)	0.028 (0.052)	0.028 (0.052)
HDL chol. (mmol/l)	−0.453 (0.102)	−0.427 (0.275)	−0.637 (0.134)	−0.600 (0.110)	−0.473 (0.187)	−0.458 (0.187)	−0.477 (0.187)	−0.464 (0.187)

(b) Nested case-control study within the full cohort								

	Complete-case	MI full-cohort approach		MI substudy approach		MI Matched set	MI intermediate approach	
					
		MI-approx	MI-SMC	MI-approx	MI-SMC		MI-approx	MI-SMC
	Est (SE)	Est (SE)	Est (SE)	Est (SE)	Est (SE)	Est (SE)		

Sex (baseline=male)	0.522 (0.195)	−0.435 (0.077)	−0.411 (0.076)	−0.523 (0.123)	−0.501 (0.118)	−0.508 (0.120)	−0.509 (0.122)	−0.510 (0.122)
Age (years)	0.114 (0.018)	0.096 (0.006)	0.097 (0.006)	0.106 (0.011)	0.111 (0.011)	0.111 (0.011)	0.105 (0.011)	0.104 (0.011)
Education*: 1	ref							
Education: 2	−0.068 (0.280)	−0.087 (0.098)	−0.084 (0.106)	−0.082 (0.197)	−0.129 (0.183)	−0.114 (0.188)	−0.107 (0.210)	−0.086 (0.201)
Education: 3	−0.269 (0.275)	−0.354 (0.103)	−0.356 (0.109)	−0.402 (0.195)	−0.461 (0.185)	−0.454 (0.186)	−0.418 (0.205)	−0.399 (0.203)
Non-smoker	ref							
Current smoker	0.806 (0.198)	0.595 (0.072)	0.600 (0.078)	0.829 (0.135)	0.806 (0.130)	0.795 (0.131)	0.811 (0.138)	0.803 (0.137)
White race	ref							
Non-white race	0.415 (0.209)	0.525 (0.077)	0.529 (0.078)	0.390 (0.146)	0.424 (0.135)	0.421 (0.136)	0.376 (0.140)	0.375 (0.145)
SBP (mmHG)	0.010 (0.005)	0.014 (0.002)	0.015 (0.002)	0.015 (0.003)	0.014 (0.003)	0.015 (0.003)	0.015 (0.003)	0.015 (0.003)
BMI (kg/metres^2^)	0.063 (0.017)	0.038 (0.007)	0.037 (0.006)	0.057 (0.012)	0.055 (0.012)	0.055 (0.011)	0.056 (0.012)	0.056 (0.012)
Total chol. (mmol/l)	0.039 (0.081)	0.032 (0.043)	0.031 (0.037)	−0.034 (0.051)	0.001 (0.049)	0.006 (0.049)	−0.034 (0.051)	−0.034 (0.051)
HDL chol. (mmol/l)	−0.302 (0.212)	−0.570 (0.132)	−0.664 (0.135)	−0.493 (0.146)	−0.458 (0.140)	−0.471 (0.139)	−0.502 (0.145)	−0.502 (0.145)
