# Supplementary material for: Multiple Imputation of Missing Data in Nested Case-Control and Case-Cohort Studies
Source: Biometrics. Author manuscript; Available in PMC 2019 Apr 24. (PMC6481559; doi:10.1111/biom.12910)

Web-based Supplementary Materials for:

Multiple imputation of missing data in nested case-control and case-cohort studies

by

Ruth H. Keogh[1,*], Shaun R. Seaman[2], Jonathan W. Bartlett[3], and Angela M. Wood[4]

[1]Department of Medical Statistics, London School of Hygiene and Tropical Medicine, London, UK.

[2]MRC Biostatistics Unit, Cambridge, UK.

[3]Statistical Innovation Group, AstraZeneca, Cambridge, UK.

[4]Department of Public Health and Primary Care, University of Cambridge, Cambridge, UK.

**email:* Ruth.keogh@lshtm.ac.uk

S1. Missingness in several variables

Section 2 of the main text outlined the MI methods for Cox regression in a full-cohort setting for a single covariate with missing data. Often there are missing data in several covariates, say $X = (X_1, X_2, \ldots, X_p)'$, and missingness in these variables may be non-monotone. There are two main ways of handling missing data in more than one variable: 'joint modelling' and 'full conditional specification' (FCS) (also called 'multiple imputation by chained equations'). We focus here on FCS, because both of the MI methods considered in this paper (MI-approx and MI-SMC) easily extend to handle missingness in several variables using this method. In the FCS method, missing values of X_k $(k = 1, \ldots, p)$ are drawn from the distribution $p(X_k|T, D, Z, X_{-k})$ where $X_{-k} = (X_1, \ldots, X_{k-1}, X_{k+1}, \ldots, X_p)'$. An iterative procedure is used in which the previous imputed value of X_{-k} is used to impute X_k until the process has converged to a stationary distribution.

For MI-approx, the FCS algorithm to generate a single imputed dataset is as follows.

(1) Replace the missing values in X by arbitrary starting values, to create a complete data set. In practice, one could replace missing values of X_k $(k = 1, \ldots, p)$ by the mean of X_k among those individuals in whom X_k is observed. Set $k = 1$.

(2) If X_k is a continuous variable, fit the imputation model $X_k = \alpha_0 + \alpha_1' Z + \alpha_2 D + \alpha_3 \widehat{H}(T) + \alpha_4' X_{-k} + \epsilon$ with residual error variance σ_ϵ^2 to the subset of individuals for whom X_k is observed, using the current values of X_{-k}. If X_k is a binary variable, the imputation model is the logistic regression logit $P(X_k = 1) = \alpha_0 + \alpha_1' Z + \alpha_2 D + \alpha_3 \widehat{H}(T) + \alpha_4' X_{-k}$. Take a random draw $(\alpha_0^*, \alpha_1^*, \alpha_2^*, \alpha_3^*, \alpha_4^*, \sigma_\epsilon^{2*})$ (if X_k is continuous) or $(\alpha_0^*, \alpha_1^*, \alpha_2^*, \alpha_3^*, \alpha_4^*)$ (if X_k is binary) from the approximate posterior distribution of the parameters in this model.

(3) If X_k is continuous, then for each individual with missing X_k in the original data set, replace the current value of X_k with a sample from a normal distribution with mean

$\alpha_0^* + \alpha_1^{*\prime} Z + \alpha_2^* D + \alpha_3^* \widehat{H}(T) + \alpha_4^{*\prime} X_{-k}$ and variance σ_ϵ^{2*}. If X_k is binary, sample instead from a Bernoulli distribution with the same mean, i.e. $\alpha_0^* + \alpha_1^{*\prime} Z + \alpha_2^* D + \alpha_3^* \widehat{H}(T) + \alpha_4^{*\prime} X_{-k}$.

(4) If $k < p$, set $k = k + 1$ and return to step 2.

Repeat steps 2–4 until the sampled values of X converge in distribution. At this point, use these sampled values as the imputed values for the single imputed dataset. Repeat the whole process M times to generate M imputed datasets.

S2. Substantive model compatible multiple imputation ('MI-SMC')

Here we describe the MI-SMC method referred to in Section 2.3 of the main text. For MI-SMC with p partially observed variables, the algorithm to generate one imputed data set is as follows.

(1) Replace the missing missing values in X with arbitrary starting values, to create a complete dataset. Set $k = 1$.

(2) Fit the Cox model to the current complete data set to obtain estimates $(\hat\beta_X, \hat\beta_Z)$ and their estimated variance $\widehat{\Sigma}$. Draw values β_X^*, β_Z^* from a joint normal distribution with mean $(\hat\beta_X, \hat\beta_Z)$ and variance $\widehat{\Sigma}$.

(3) Calculate Breslow's estimate, denoted $H_0^*(t)$, of the baseline cumulative hazard $H_0(t)$ using the parameter values β_X^*, β_Z^* and the current imputations of X.

(4) Fit a regression model (e.g. linear or logistic, as appropriate) of X_k on X_{-k} and Z to the current complete data set. Draw a value γ_{Xk}^* from the approximate joint posterior distribution of the parameters γ_{Xk} in this model.

(5) For each individual for whom X_k is missing, (a) draw a value X_k^* from the distribution $p(X_k|X_{-k}, Z; \gamma_{Xk}^*)$ and let X^* denote X with X_k replaced by its proposed value X_k^*, (b) draw a value U from a uniform distribution on $[0, 1]$, and (c) accept the proposal X_k^* if either $D = 0$ and $U \leqslant \exp\{-H_0^*(t) e^{\beta_X^* X^* + \beta_Z^{*\prime} Z}\}$ or $D = 1$ and $U \leqslant H_0^*(t) \exp\{1 + \beta_X^* X^* +$

$\beta_Z^{*\prime} Z - H_0^*(t) e^{\beta_X^* X^* + \beta_Z^{*\prime} Z}\}$. If X_k^* is not accepted, then discard it and repeat (a), (b) and (c).

(6) If $k < p$, let $k = k + 1$ and return to step 2.

Repeat steps 2–6 until the sampled values of X converge in distribution. At this point, use these sampled values as the imputed values for the single imputed dataset. Repeat the whole process M times to generate M imputed datasets.

In the full-cohort and intermediate MI approaches, the MI-SMC algorithm just described is applied to all the data on the full cohort. In the setting described in the main text, there were only $p = 2$ partially observed variables, X_1 and X_2 (as well as the fully observed variables Z), but the algorithm allows for any number of partially observed variables.

In the substudy approach, the MI-SMC algorithm is applied only to the data on the substudy, but with the following modifications to steps 3 and 4 (steps 1, 2, 5 and 6 remain unchanged). Steps 3 and 4 involve estimating, respectively, the (population) baseline cumulative hazard $H_0(t)$ and the parameters of the distribution $p(X_2|X_1, Z)$. In Section 5.2 of the main text we described modified estimators of these two quantities that should be used in steps 3 and 4 when using the substudy approach. In the setting described in the main text, there was only $p = 1$ partially observed variable, X_2 (since X_1 is fully observed in the substudy), but the algorithm allows for any number of partially observed variables.

S3. Further details of the simulation study

We base our simulation partly on information from a recent review of case-cohort studies (Sharp et al., 2014) and partly on studies in cardiovascular epidemiology, from which we take our example in Section 7 of the main text. The full cohort size of 15,000 individuals used in the simulation study was approximately the 25th percentile of full cohort sizes in the studies reviewed by Sharp et al. (2014). Variable Z is binary and was generated from a

Bernoulli distribution with probability 0.5, variable X_2 is binary and was generated from a Bernoulli distribution such that $logitPr(X_2 = 1|Z) = 0.5Z$, and variable X_1 is continuous and was generated from a normal distribution with mean $0.25Z + 0.25X_2$ and variance 1.

Loss-to-follow-up times were generated from an exponential distribution with hazard λ_C. The parameter λ in the hazard model used to generate event times, and λ_C, were chosen so that 10% of individuals have the event, 20% are lost to follow-up and the remainder are administratively censored after 15 years of follow-up. The follow-up time and event rate are realistic values for studies in cardiovascular epidemiology. For the case-cohort sample we used a subcohort sampling percentage of 5%, which was similar to the median subcohort sampling fraction of 4.1% found in the review by Sharp et al. (2014)

Missingness in X_2 was generated using probability of missingness $\exp(a + 0.2Z + 0.2D + 0.2ZD)/\{1+\exp(a+0.2Z+0.2D+0.2ZD)\}$, with a chosen to give 10% or 50% missingness.

All MI analyses used 10 imputed data sets. MI-SMC used 100 iterations; using fewer iterations results in non-convergence for a situation with large effect size and large amounts of missing data when using the full-cohort approach in the case-cohort setting, though in other simulation scenarios fewer iterations (e.g. 10) tended to be sufficient. In the nested case-control setting, the MI-SMC full-cohort approach did not require a large number of iterations because a larger proportion of the full cohort is in the nested case-control sample. MI-approx analyses used 5 iterations, which is the default in the mice package in R.

S4. Additional simulation scenario: Model misspecification

We investigated the performance of the methods when the imputation model is misspecified. For this, X_1 was generated from a log normal distribution with $\log X_1$ having mean $0.25Z$ and standard deviation 0.65 (so that the variance of X_1 was around 1, as in the main simulation). X_2 was generated from a Bernoulli distribution using logit $Pr(X_2 = 1|X_1, Z) = 0.5Z + 0.25(X_1 + X_1^2)$. In MI-approx, the misspecified imputation model for X_1 (full cohort

and intermediate approaches) was $X_1 = \alpha_0 + \alpha_1 X_2 + \alpha_2' Z + \alpha_3 D + \alpha_4 \widehat{H}(T) + \epsilon$, and the misspecified imputation model for X_2 was logit $Pr(X_2 = 1|X_1, Z, T, D) = \alpha_0 + \alpha_1 X_1 + \alpha_2' Z + \alpha_3 D + \alpha_4 \widehat{H}(T)$. MI-SMC allows imputed variables to be used on a transformed scale in the substantive model. We considered two forms for the model for $X_1|X_2, Z$: (1) a misspecified normal distribution for $X_1|X_2, Z$ with main effects of X_2 and Z; (2) a correctly specified normal distribution for $\log X_1|X_2, Z$ with main effects of X_2 and Z. In both cases we used a model for $X_2|X_1, Z$ with main effects of X_1 and Z, which is misspecified.

S5. Software

Traditional analyses of nested case-control and case-cohort studies (Section 3 of the main text) can be performed in standard software using Cox regression. For case-cohort studies the data should be modified so that cases not in the subcohort have a start of follow-up time which is just before their event time. For nested case-control studies the Cox regression should be stratified by the matched set indicator (this gives an analysis which is equivalent to using conditional logistic regression).

Example R and Stata code for applying the methods described in this paper is available at https://github.com/ruthkeogh/MI-CC.

MI-approx (full-cohort, intermediate, and substudy approaches) can be implemented using the `mice` package in R (Van Buuren and Groothuis-Oudshoorn, 2011) and the `mi` command in Stata. The MI-approx analyses could also be performed using other statistical packages (e.g. `PROC MI` in SAS). The MI-approx methods described in this paper require estmates of the cumulative hazard (see https://github.com/ruthkeogh/MI-CC).

For the full-cohort and intermediate approaches, MI-SMC is applied in the full cohort and this can be implemented using the `smcfcs` package (Bartlett and Morris, 2015) in Stata (see https://github.com/jwb133/Stata-smcfcs, and also available on SSC) or R (see

https://github.com/jwb133/smcfcs, and also available on CRAN). We have implemented the MI-SMC substudy approach (main text Section 5.2) in the new `smcfcs.nestedcc` and `smcfcs.casecohort` functions in the `smcfcs` package in R.

MI matched set (Section 5.3 of the main text) can be performed using `mice` in R or `ice` in Stata (Royston, 2005) after some rearrangement of the data, as described by Seaman and Keogh (2015). MI matched set could also be performed using other statistical packages (e.g. `PROC MI` in SAS).

S6. Extensions

S6.1 *Censoring, left-truncation and auxiliary variables*

MI-approx and MI-SMC (Section 2 of the main text) assume that any right-censoring occurs independently of the variables with missing data given the fully observed variables. Such dependence can be accommodated in MI-approx by adding the term $\widehat{H}_{\mathsf{cens}}(t)$ into the imputation model, where $\widehat{H}_{\mathsf{cens}}(t)$ denotes the Nelson-Aalen estimate of the cumulative hazard for the censoring (Borgan and Keogh, 2015). Event times are also commonly subject to left-truncation, for example when the time scale for the analysis is age but individuals are followed from age at entry to the cohort. When there is left truncation, the distribution we wish to draw imputed values from becomes $p(X|T, D, Z, T \geqslant T_L)$, where T_L is the left-truncation time. Left-truncation can be accommodated in MI-approx by replacing $\widehat{H}(t)$ with $(\widehat{H}(t) - \widehat{H}(t_L))$ and $\widehat{H}_{\mathsf{cens}}(t)$ by $(\widehat{H}_{\mathsf{cens}}(t) - \widehat{H}_{\mathsf{cens}}(t_L))$. These methods can be applied directly in the full-cohort approach and intermediate approach for nested case-control and case-cohort studies. For the substudy approach, $\widehat{H}_{\mathsf{cens}}(t)$ can be obtained in the maximum information setting and the time-only information setting using the data on (T, D) in the full cohort. In the minimum information setting in a case-cohort study $H_{\mathsf{cens}}(t)$ can be estimated using a modified version of $\widehat{H}^*_{CC}(t)$ with 'event' replaced by 'censoring'. MI-SMC has been

extended to allow competing risks (Bartlett and Taylor, 2016) and this can be used to handle dependence of right-censoring on variables with missing data. Left truncation has also been incorporated in the the Stata version of the `smcfcs` package.

Auxiliary variables are variables that may be predictive of the value of a partially missing variable but that we do not wish to include as covariates in the substantive model. Auxiliary variables could be incorporated into imputation models in all of the MI methods with no other changes, except that in MI-SMC the auxiliary variables are included in the outcome model at the imputation stage but then omitted from the analysis model.

S6.2 *Matching, weighting, and stratification*

In nested case-control studies, controls are often matched to cases using variables observed in the full cohort. Matching variables should be included as additional predictors in the imputation model in MI-approx. In MI-SMC they should be included in the covariate model used for the proposal distribution and in the Cox model. The denominator of the cumulative hazard estimate $\widehat{H}_0^{\mathrm{NCC}}(t) = \sum_{\tau_k \leqslant t} \frac{1}{\sum_{l \in \tilde{R}_k} \{n(\tau_k)/(c(\tau_k)+1)\} \exp(\hat{\beta}_{X1} X_{1l} + \hat{\beta}_{X2} X_{2l} + \hat{\beta}_Z Z_l)}$ should be modified to incorporate stratum-specific weights. In the full-cohort approach, the Cox analysis performed on the imputed data should incorporate the matching variables, either by stratifying or by including them as covariates.

The traditional case-cohort analysis (Section 3 of the main text) was described by Prentice (1986). Modifications have been proposed in which the denominator in the pseudo-partial likelihood L_{substudy} is modified to include individual weights (Onland-Moret et al., 2007). This is relevant for the substudy and intermediate approaches, in which the imputation methods can be used without modification. Case-cohort studies can also use stratified random sampling of the subcohort. One version of the traditional analysis uses a modified version of the psuedo-partial likelihood L_{substudy} in which $\tilde{R}_j$ is replaced by $\tilde{R}_{gj}$, the subset of $\tilde{R}_j$ that is in the same stratum g as the case whose event time is τ_j. See Borgan et al. (2000) for

alternative methods for use when the subcohort is selected by stratified random sampling. In MI-approx the imputation model should include the stratifying variables, and the cumulative hazard estimate $\widehat{H}_{\text{CC}}(t) = \frac{n_S(0)}{n} \sum_{\tau_k \leqslant t} \frac{d(\tau_k)}{n_S(\tau_k)}$ should be modified to incorporate stratum-specific weights in the denominator. In MI-SMC the covariate model used for the proposal distribution should include the matching variables as predictors, and the denominator of the cumulative baseline hazard estimate $\widehat{H}_0^{\text{CC}}(t) = \frac{n_S(0)}{n} \sum_{\tau_k \leqslant t} \frac{1}{\sum_{l \in S_k} \exp(\hat{\beta}_{X1} X_{1l} + \hat{\beta}_{X2} X_{2l} + \hat{\beta}_Z Z_l)}$ is modified using stratum-specific weights.

References

Bartlett, J. and Morris, T. (2015). Multiple imputation of covariates by substantive-model compatible fully conditional specification. Stata J. **15,** 437–456.

Bartlett, J. and Taylor, J. (2016). Missing covariates in competing risks analysis. Biostatistics page doi:10.1093/biostatistics/kxw019.

Borgan, O. and Keogh, R. (2015). Nested case-control studies: Should one break the matching? Lifetime Data Anal. **21,** 517–541.

Borgan, O., Langholz, B., Samuelsen, S., Goldstein, L., and Pogoda, J. (2000). Exposure stratified case-cohort designs. Lifetime Data Anal. **6,** 39–58.

Onland-Moret, N., van der A, D., van der Schouw, Y., Buschers, W., Elias, S., van Gils, C., Koerselman, J., Roest, M., Grobbee, D., and P.H.M., P. (2007). Analysis of case-cohort data: A comparison of different methods. J. Clin. Epidemiol. **60,** 350–355.

Prentice, R. (1986). A case-cohort design for epidemiologic cohort studies and disease prevention trials. Biometrika **73,** 1–11.

Royston, P. (2005). Multiple imputation of missing values: update. Stata J. **2,** 188–201.

Seaman, S. and Keogh, R. (2015). Handling missing data in matched case-control studies using multiple imputation. Biometrics **71,** 1150–1159.

Sharp, S., Poulaliou, M., Thompson, S., I.R., W., and Wood, A. (2014). A review of published analyses of case-cohort studies and recommendaions for future reporting. PLoS ONE **9,** doi:10.1371/journal.pone.0101176.

Van Buuren, S. and Groothuis-Oudshoorn, K. (2011). mice: Multivariate imputation by chained equations in R. J. Stat. Softw. **45,** 1–67.

Figure S1. Simulation study results: case-cohort study within a cohort; 10% missing X_2. The points are the means of the point estimates from 1000 simulated data sets. The horizontal lines around each point are the 95% confidence intervals obtained based on Monte Carlo errors. The relative efficiency is relative to the complete-data sub-study analysis.

(a) $\beta_{X1} = \beta_{X2} = \beta_Z = 0.2$

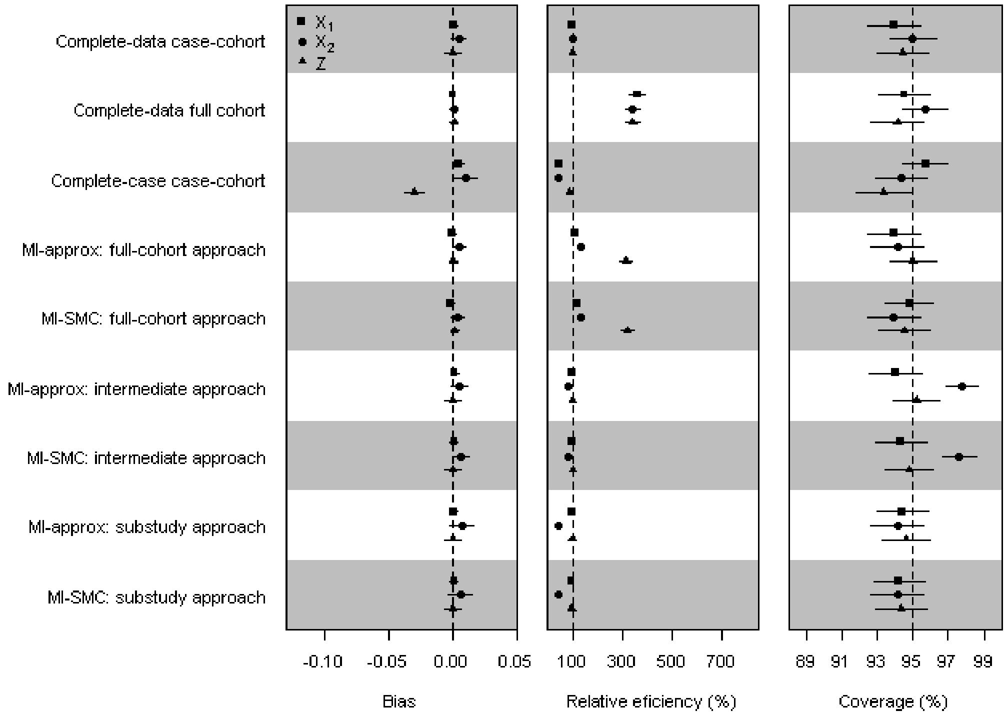

(b) $\beta_{X1} = \beta_{X2} = \beta_Z = 0.7$

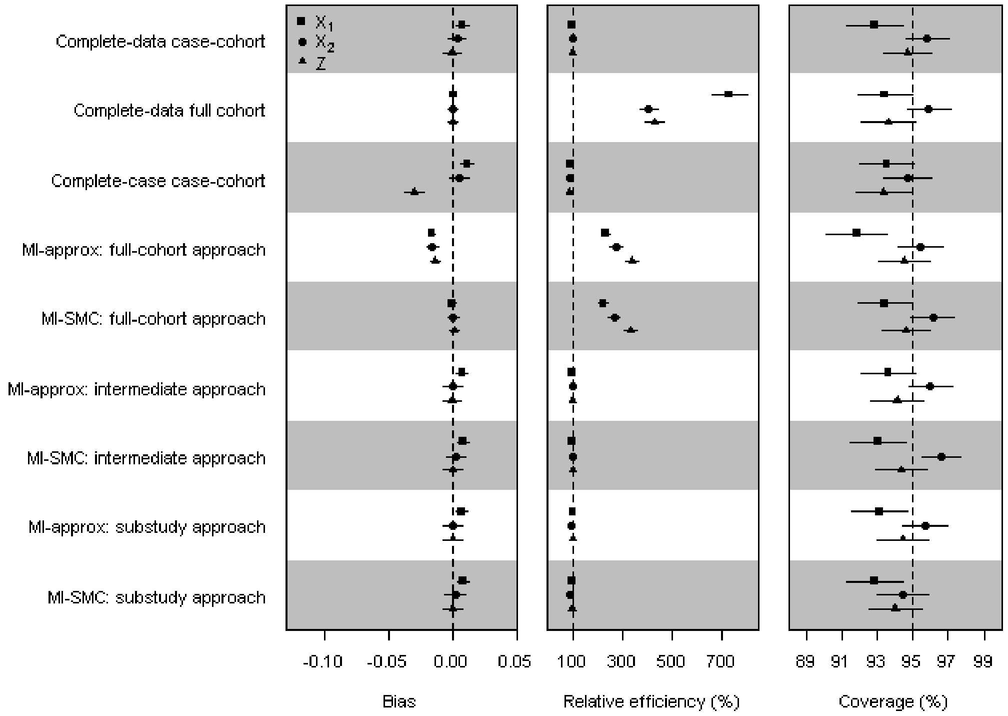

Figure S2. Simulation study results: nested case-control study with 1 control per case within a cohort; 10% missing X_2. The points are the means of the point estimates from 1000 simulated data sets. The horizontal lines around each point are the 95% confidence intervals obtained based on Monte Carlo errors. The relative efficiency is relative to the complete-data sub-study analysis.

(a) $\beta_{X1} = \beta_{X2} = \beta_Z = 0.2$

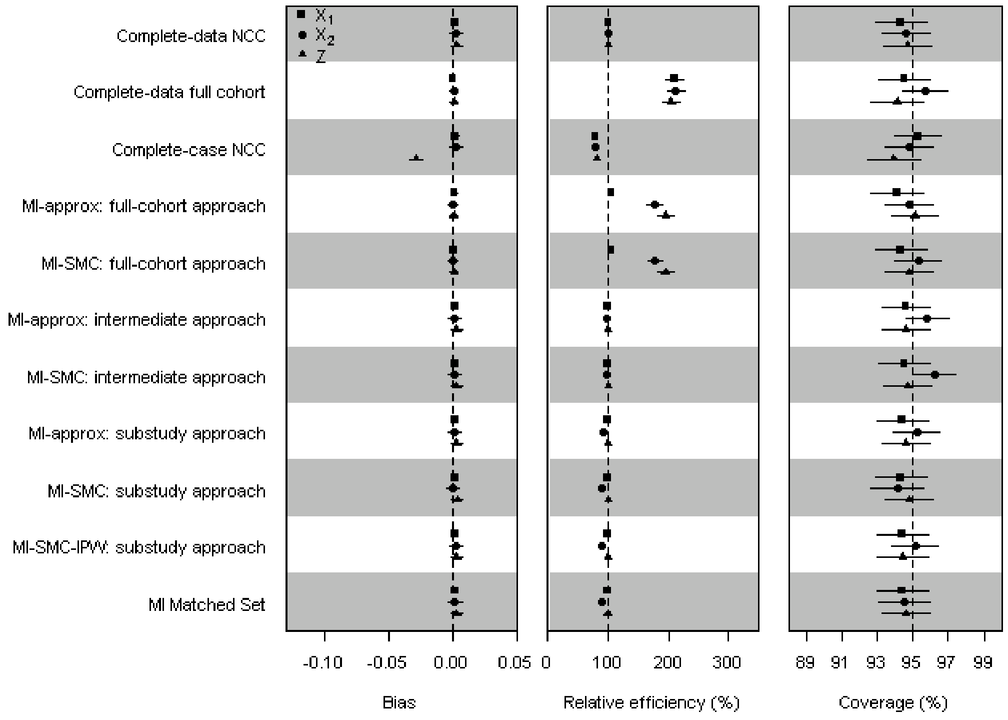

(b) $\beta_{X1} = \beta_{X2} = \beta_Z = 0.7$

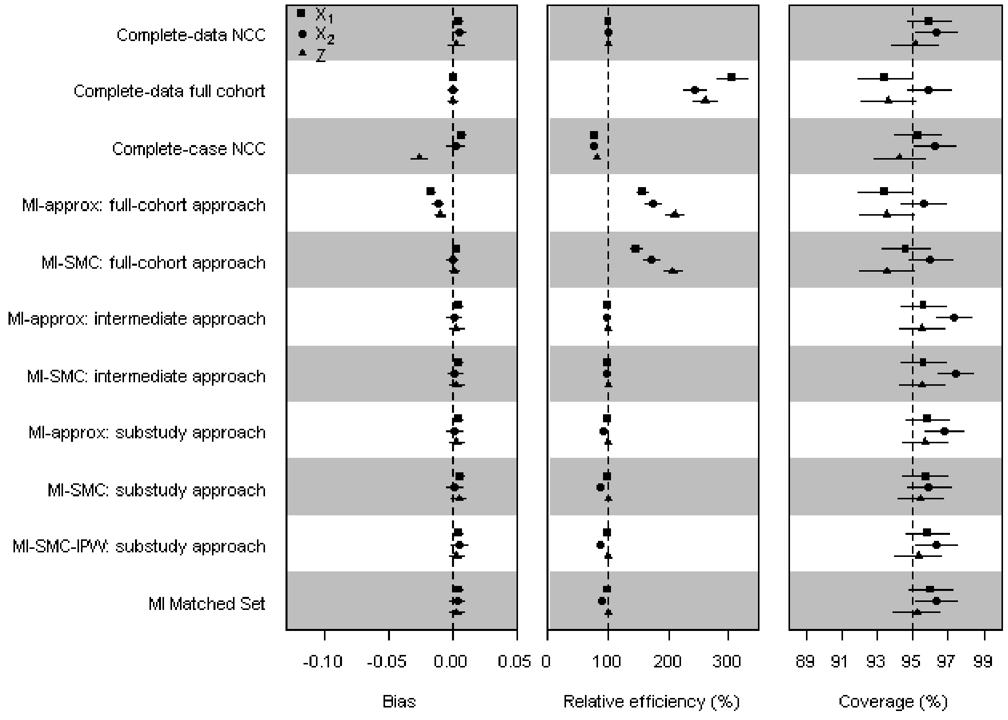

Figure S3. Simulation study results: nested case-control study with 4 controls per case within a cohort; 50% missing X_2. The points are the means of the point estimates from 1000 simulated data sets. The horizontal lines around each point are the 95% confidence intervals obtained based on Monte Carlo errors. The relative efficiency is relative to the complete-data sub-study analysis.

(a) $\beta_{X1} = \beta_{X2} = \beta_Z = 0.2$

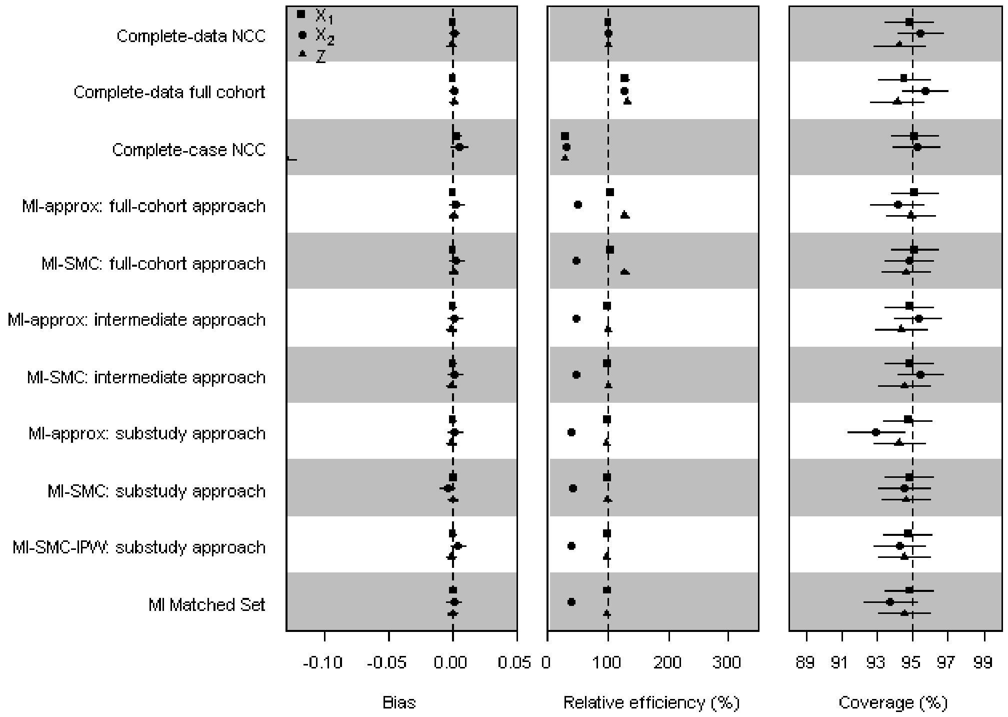

(b) $\beta_{X1} = \beta_{X2} = \beta_Z = 0.7$

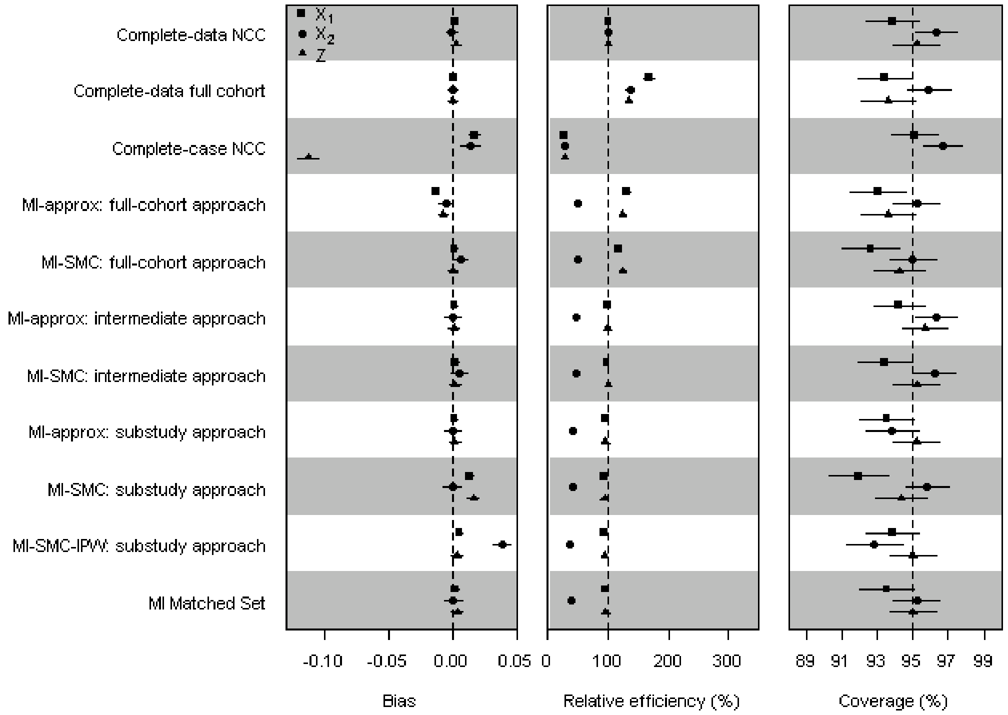

Figure S4. Simulation study results: nested case-control study with 4 controls per case within a cohort; 10% missing X_2. The points are the means of the point estimates from 1000 simulated data sets. The horizontal lines around each point are the 95% confidence intervals obtained based on Monte Carlo errors. The relative efficiency is relative to the complete-data sub-study analysis.

(a) $\beta_{X1} = \beta_{X2} = \beta_Z = 0.2$

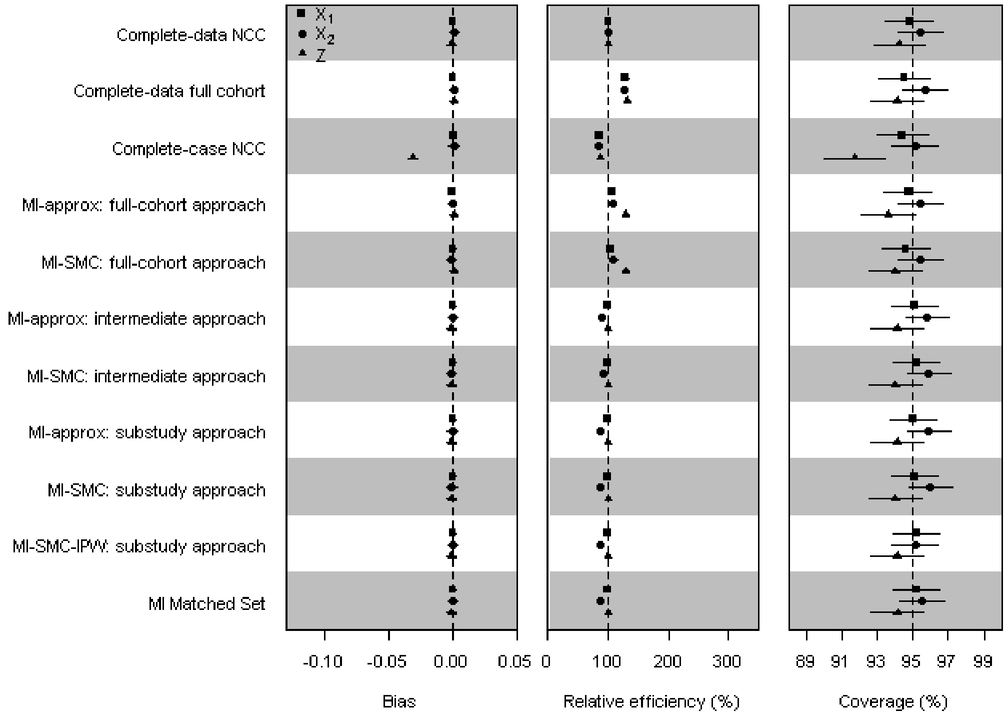

(b) $\beta_{X1} = \beta_{X2} = \beta_Z = 0.7$

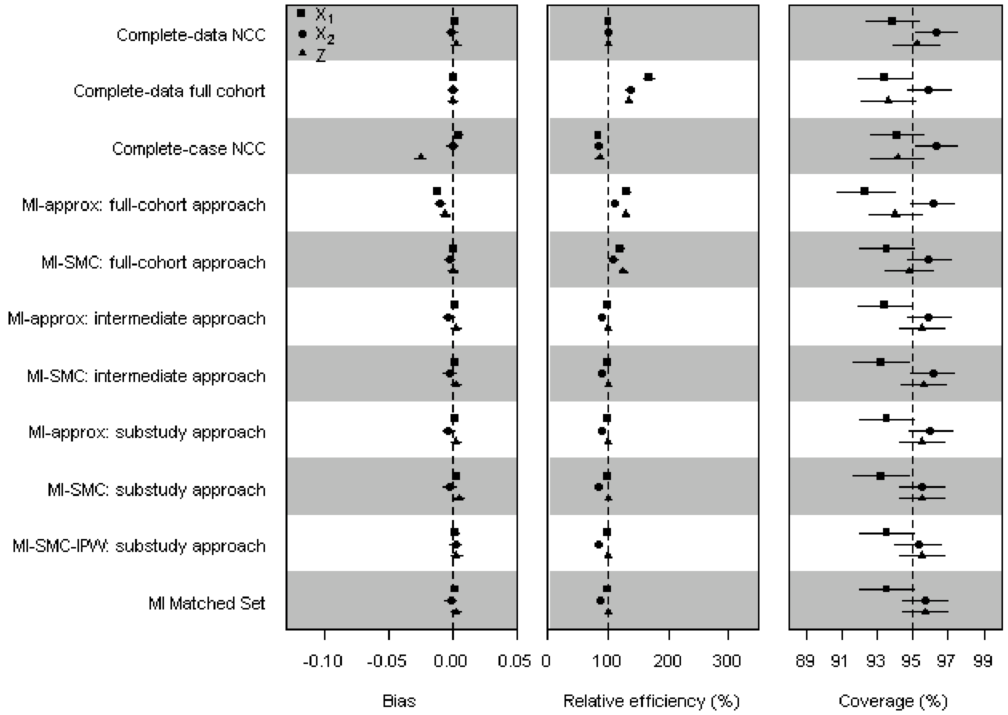

Figure S5. Simulation study results: case-cohort study within a cohort with 50% missing X_2. Results for different sample sizes and size of subcohort. The points are the means of the point estimates from 1000 simulated data sets. Horizontal lines around each point are the 95% confidence intervals obtained based on Monte Carlo errors. The relative efficiency is relative to the complete-data substudy analysis.

(a) Cohort of size 30,000 with subcohort of size 1500 (5%). $\beta_{X1} = \beta_{X2} = \beta_Z = 0.7$.

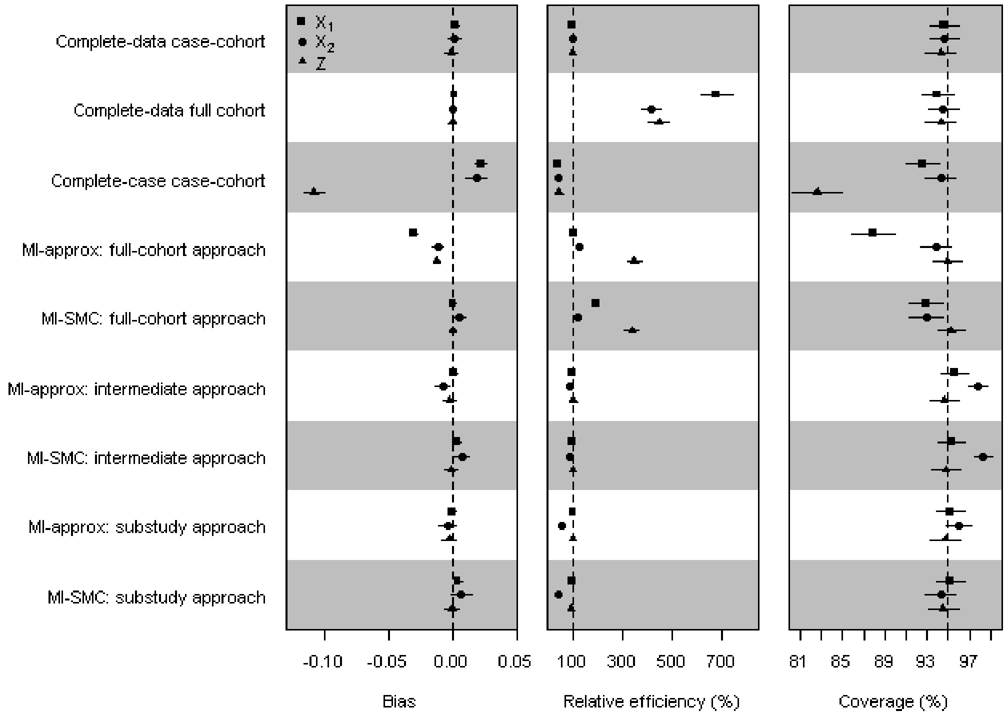

(b) Cohort of size 15,000 with subcohort of size 3000 (20%). $\beta_{X1} = \beta_{X2} = \beta_Z = 0.7$

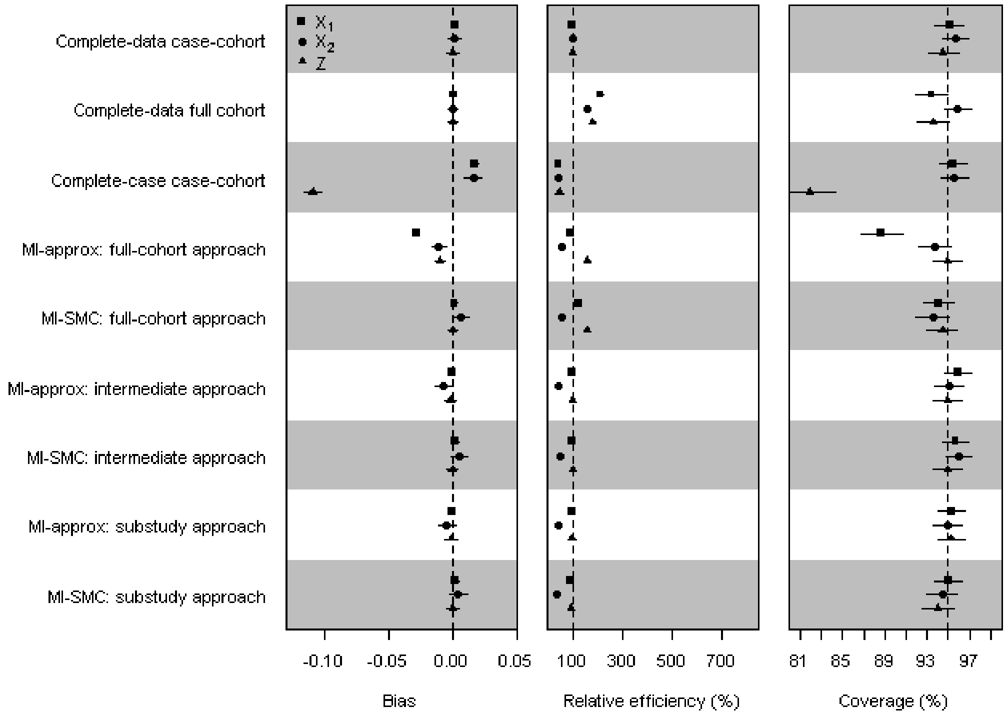

Supplement: Supplementary information [file NIHMS82524-supplement-Supplementary_information.pdf]
